# Geriatric assessment for older people with cancer: policy recommendations

**DOI:** 10.1186/s41256-023-00323-0

**Published:** 2023-09-01

**Authors:** P. A. L. Seghers, Shabbir M. H. Alibhai, Nicolò Matteo Luca Battisti, Ravindran Kanesvaran, Martine Extermann, Anita O’Donovan, Sophie Pilleron, Anna Rachelle Mislang, Najia Musolino, Kwok-Leung Cheung, Anthony Staines, Charis Girvalaki, Pierre Soubeyran, Johanneke E. A. Portielje, Siri Rostoft, Marije E. Hamaker, Dominic Trépel, Shane O’Hanlon

**Affiliations:** 1 Department of Geriatric Medicine, Diakonessenhuis, 3582 KE Utrecht, The Netherlands; 2 Department of Medicine, University Health Network, Toronto, ON M5G 2C4 Canada; 3 Department of Medicine, University of Toronto, Toronto, ON M5G 2C4 Canada; 4 Institute of Health Policy, Management, and Evaluation, University of Toronto, Toronto, ON M5G 2C4 Canada; 5 Department of Medicine, The Royal Marsden NHS Foundation Trust, Downs Road, Sutton, London, SM2 5PT UK; 6 Breast Cancer Research Division, The Institute of Cancer Research, 15 Cotswold Road, Sutton, London, SM2 5NG UK; 7 National Cancer Centre Singapore, Singapore, Singapore; 8 Department of Oncology, Moffitt Cancer Center, University of South Florida, Tampa, FL USA; 9 Applied Radiation Therapy Trinity (ARTT), School of Medicine, Trinity College Dublin, Dublin, Ireland; 10 Nuffield Department of Population Health, Big Data Institute, University of Oxford, Oxford, UK; 11 Department of Medical Oncology, Flinders Centre for Innovation in Cancer, College of Medicine and Public Health, Flinders University, Bedford Park, SA 5042 Australia; 12 International Society of Geriatric Oncology (SIOG), International Environmental House 2, Chemin de Balexert 7-9, 1219 Chatelaine, Switzerland; 13 School of Medicine, University of Nottingham, Nottingham, UK; 14 School of Nursing, Psychotherapy and Community Health, Dublin City University, Dublin, Ireland; 15 European Network for Smoking and Tobacco Prevention (ENSP), Brussels, Belgium; 16 Department of Medical Oncology, Institut Bergonié, Inserm U1312, SIRIC BRIO, Université de Bordeaux, 33076 Bordeaux, France; 17 Department of Medical Oncology, Leiden University Medical Center-LUMC, 2333 ZA Leiden, The Netherlands; 18 Department of Geriatric Medicine, Oslo University Hospital, 0424 Oslo, Norway; 19 Institute of Clinical Medicine, University of Oslo, 0318 Oslo, Norway; 20 Global Brain Health Institute, Trinity College Dublin, The University of Dublin, Dublin, Ireland; 21 Trinity Institute of Neurosciences, School of Medicine, Trinity College Dublin, Dublin, Ireland; 22 Department of Geriatric Medicine, St Vincent’s University Hospital, Dublin, D04 T6F4 Ireland; 23 Department of Geriatric Medicine, University College Dublin, Dublin, D04 V1W8 Ireland

**Keywords:** Geriatric assessment, Aged, 80 and over, Decision making, Shared, Neoplasms, Quality of life, Survival, Toxicity, Health policy, Medical oncology

## Abstract

Most cancers occur in older people and the burden in this age group is increasing. Over the past two decades the evidence on how best to treat this population has increased rapidly. However, implementation of new best practices has been slow and needs involvement of policymakers. This perspective paper explains why older people with cancer have different needs than the wider population. An overview is given of the recommended approach for older people with cancer and its benefits on clinical outcomes and cost-effectiveness. In older patients, the geriatric assessment (GA) is the gold standard to measure level of fitness and to determine treatment tolerability. The GA, with multiple domains of physical health, functional status, psychological health and socio-environmental factors, prevents initiation of inappropriate oncologic treatment and recommends geriatric interventions to optimize the patient’s general health and thus resilience for receiving treatments. Multiple studies have proven its benefits such as reduced toxicity, better quality of life, better patient-centred communication and lower healthcare use. Although GA might require investment of time and resources, this is relatively small compared to the improved outcomes, possible cost-savings and compared to the large cost of oncologic treatments as a whole.

## Background

**Fig. 1:**
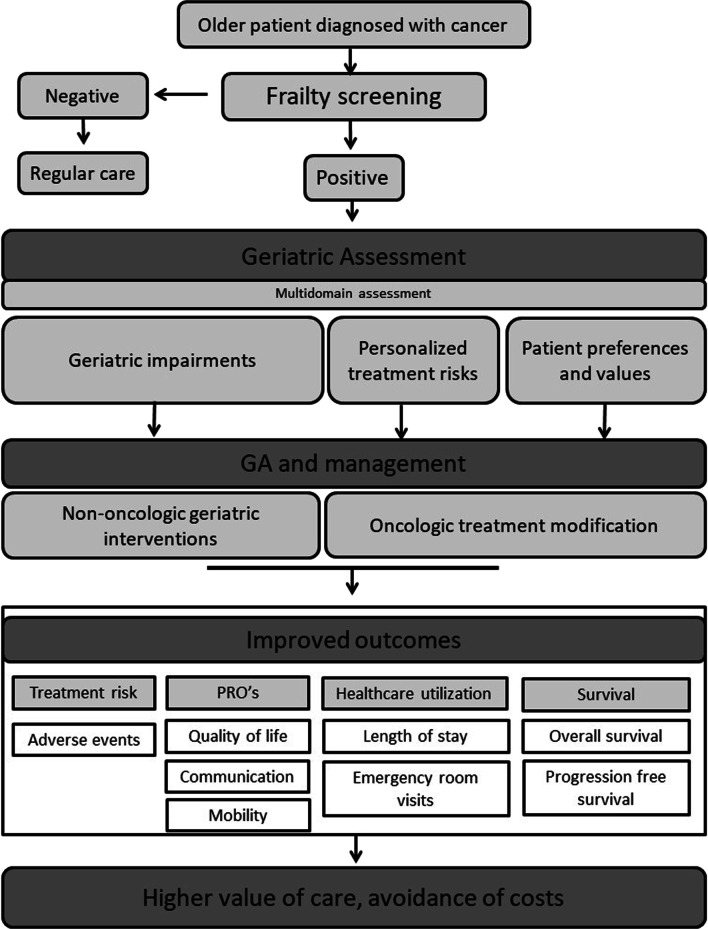
Geriatric oncology in a nutshell. Flow chart of how the different components of geriatric oncology impact the outcomes and thus value of care and costs

Cancer is predominantly a disease of ageing, as over half of all cancer cases occur in people over 65 years and this proportion is expected to increase in the coming decades as well as the older population itself that is expected to double by 2050 [1, 2]. Older patients are commonly under-represented in cancer clinical trials that inform standard treatment decisions, thus cancer clinical guidelines might not be applicable to this population [3]. The result is that older adults with cancer are at risk of inappropriate treatment, and have a higher risk of complications and toxicity [4, 5]. Although expenditure on treatment has exploded for most types of cancer, survival in older adults with cancer has hardly improved at all [6]. It is against this backdrop that the field of geriatric oncology was established, to improve access to appropriate treatments, tailor treatment to the individual patient and ensure better outcomes for older adults with cancer (Fig. 1). The geriatric oncology approach is recommended as the standard of care for this group of individuals by international consensus and guidelines [7, 8]. The aim of this paper is to share the clinical rationale for geriatric oncology with policymakers and make recommendations for development of these services [7].

Older patients are a heterogeneous group and require personalised care and treatment approaches. One older patient may be highly active and fit, whereas another may be living with frailty and require help for daily activities such as preparing a meal or going outside. Frailty is an often-used concept to describe whether a patient is fit or vulnerable. It describes a state of increased vulnerability which results in a reduced ability to respond to a stressor (like an illness or a burdensome treatment) which in turn increases the risk of adverse outcomes [9]. With ageing, frailty becomes more common, but the relationship between age and frailty is complex and not necessarily linear [9]. In older patients biological age (meaning general fitness or frailty), and not chronological age determines treatment tolerance.

In addition, older patients commonly have other diseases (comorbidities) and often have more than one major disease (multimorbidity). This also affects how their body will respond to cancer and oncologic treatments and is therefore important to incorporate into care and decision-making [10, 11]. Unfortunately, most health systems focus on single diseases, which is a poor fit for the complex needs of older people. Older people are not only heterogeneous from a medical point of view, but also when it comes to their values and priorities in outcomes of treatment. Traditionally, the effect of oncologic treatments has been measured by survival and toxicity rates. However, many older patients prioritize quality of life or maintaining independence over survival [12]. These other non-traditional outcomes, sometimes referred to as patient related outcomes (PROs), and the effect of oncologic treatment on these outcomes, are equally important aspects in the care for, decision making with, and research about older patients with cancer.

## Assessment of fitness in older cancer patients

In geriatric oncology, the gold standard to assess an older patient’s level of frailty or fitness is a multidomain assessment, called a Geriatric Assessment (GA). GA identifies previously unknown impairments and strengths of an individual by systematically assessing multiple domains, many of which are not typically assessed by an oncologist in routine care. These domains include as a minimum functional status, mobility, nutritional status, mood, cognition and comorbidities [13, 14]. Functional status explores if a patient is independent in their activities of daily living (ADLs) such as feeding, bathing, dressing and independence in the instrumental activities of daily living (iADLs) such as shopping, preparing meals or handling finances. The mobility domain examines if there have been any falls and may include physical tests about balance and gait speed. Nutritional status deals with any weight loss or risk of weight loss. During the interview, cognition is explored and often a screening test is performed. Additionally, questions are asked to investigate mood and comorbidities. The exact tools and questionnaires used for the various domains of GA differ among countries and institutions and depend on local guidelines, patient characteristics and validation status [14].

Geriatric Assessment comprises the process of tailoring the oncologic treatment plan and addressing any impairments found with geriatric interventions to optimize the patient’s general health status, as well as following up those interventions [15, 16]. Examples of geriatric interventions are physical therapy in patients with reduced mobility, meals on wheels when patients are unable to cook for themselves and closer involvement of a caregiver in cases of cognitive decline.

Sometimes in oncology abbreviated versions of GA are performed that don’t assess all domains or take less than fifteen minutes to complete. These may serve as screening tools to select which patients require a GA to reduce the amount of resources, but are not recognised as a GA [17]. Examples of the most studied screening tools in older patients with cancer are Geriatric 8 (G8), Triage Risk Screening Tool (TRST) and Vulnerable Elders Survey-13 (VES-13). Although some have relative high sensitivity for frailty, they all have poor negative predictive value (maximum 60%) and thus do not replace GA [17, 18]. In oncology, performance scores (e.g. ECOG performance status) are used to determine if a patient is fit enough for oncologic therapy, but these fail to take into account age-related problems such as multimorbidity, frailty or cognition [19]. For the older patient a GA is a more suitable method to predict treatment tolerability.

## Benefits of GA in optimising the care of older adults with cancer

In recent years GA has been applied in the cancer setting more frequently, and convincing evidence for its use exists. GA can be used to help predict personalised treatment risk and mortality [20]. Multiple tools have been developed to predict the risk of chemotherapy toxicity in older patients by using individual items gathered from GA [21, 22]. This information leads to more accurate weighing up of benefits and risks of a proposed treatment. Additionally, GA detects impairments that are not elicited by routine history and physical examination. The prevalence of geriatric impairments found in older patients about to start oncologic treatment has been reported to be as high as 72.4%, but varies between populations [23]. Finding geriatric impairments allows for tailored interventions that can lead to increased resilience and thus better coping with the cancer and the oncologic treatments and their side effects. The outcome of GA can also be used to individualize oncologic treatment plans. In a recent systematic review the oncologic treatment plans were changed in 31% of patients (median of all studies), mostly towards a less intensive treatment option, and in studies that included a multidisciplinary team evaluation to discuss GA, these numbers were higher [24]. That GA can improve traditional treatment outcomes was shown in two randomized controlled trials, GAP 70 + and GAIN [25, 26]. Even though they used different methods to incorporate GA into the care trajectory and included different study populations, both showed a significant reduction in moderate to severe chemotherapy-related toxic effects without compromising survival [25, 26].

Furthermore, Patient-related outcomes (PROs) were found to be improved by GA, for example in the INTEGERATE study, that assessed the effect of geriatrician-lead GA, compared to usual care [27, 28]. During follow-up, the intervention group reported better quality of life [27]. Similar positive effects of GA were found for studies assessing physical functioning, such as a reduced number of falls over three months in GAP 70 + intervention [25] and on symptom burden [29]. In addition, GA guided pathways improved patient-centred and caregiver-centred communication about ageing-related problems [26, 30]. Patients were more satisfied after the first visit and satisfaction remained higher over six months follow-up [30].

Moreover, multiple studies found a decline in healthcare use in GA-driven care pathways, such as emergency department visits and unplanned hospitalisations [27, 29]. In a review on cost-effectiveness, more than 90% of the included studies showed a positive or a neutral effect on length of stay [31]. Lastly, GA can be used during follow-up of older patients with cancer to identify new impairments early [32].

GA thus helps to personalize management, which prevents inadequate treatment and improves outcomes. Overtreatment in older patients with cancer is defined as giving treatment to a patient who would not have needed treatment, or too intensive treatment in a vulnerable patient, who might have benefited more from a less intensive therapy [5]. Undertreatment is the use of a less intensive oncologic treatment in a fit older adult who would otherwise have had a greater net benefit from a more intensive treatment, or not providing geriatric interventions to optimize the patient’s general health status and increase resilience regardless of which oncologic therapy is chosen [5].

## Cost-effectiveness of geriatric assessment

Evidence on possible cost-effectiveness of GA is starting to appear and seems to disprove that GA takes too long or is too resource-intensive. A recent study showed that GA in a special oncology clinic could save 7,387 Canadian dollars per patient, the equivalent of 5,433 euros or 5704 US dollars [33]. In this clinic, GA required 1–1.5 h and was performed by a clinical nurse specialist and a geriatrician. Recommendations both for the oncologic treatment decision and interventions for the optimization of geriatric domains were given to the referring oncologist. In half of the patients, oncologic treatment plans were modified. Of those modified treatments, 96% involved reduction in treatment intensity or a change to best supportive care. In an example given, only 7% of the total costs were related to GA such as an extra clinical follow up and extra phone calls by the nurses; the other 93% of the total costs was accounted for by the oncological treatment [33]. This huge difference between the oncologic expenses and additional costs for geriatric services was mentioned before by other studies [31, 34]. The authors calculated that the overall cost-saving effect of the group would remain as long as at least 4% of all the patients received a treatment modification [33]. In this study, the effect of reduced toxicity and unplanned health care use was not even taken into account and thus savings may have been underestimated [33].

A narrative review on the cost-effectiveness of geriatric assessment in oncology found a positive or neutral effect on cost-effectiveness propensity in 28 of the 29 included studies. Most frequent positive effects were caused by reduced health care use or toxicity, but also improved management of symptoms and thus improved quality of life or with no increase in costs or a similar length of stay were discussed as cost-effective [31].

In addition to these demonstrated benefits on medical costs in one group of patients, GA may also show cost-effectiveness on a societal perspective. For example, because improved mobility may preserve independence, this can lead to lower health-care use of home care resources or less involvement of a caregiver, who in turn can work more hours. Little research on this societal impact has been conducted. In a study that estimated the economic burden of cancer and ageing that may be avoided by the proper use of GA in the US, EU4 (France, Germany, Italy, Spain), UK and China, it was calculated that 64.2 billion US dollars in the US, 48.6 billion US dollars in the European countries and United Kingdom, and 34.0 billion US dollars in China could be avoided by 2030 [35].

Direct cost-effectiveness and cost-utility studies remain scarce; one study that is currently underway is GERONTE (https://geronteproject.eu/) [36]. It is a European cluster-randomized controlled trial that will evaluate a new care pathway for older multimorbid patients with cancer which includes GA. The primary endpoint will be quality of life, but cost-effectiveness and a cost-utility analysis will be a substantial part of the study including both a societal and a payer perspective.

## Models of geriatric oncology

Multiple care models for older patients with cancer have been implemented in different countries [37]. The choice of model should be informed by the resources available to facilitate the full integration of information derived by GA into oncologic decision making to enhance the effect on outcomes. Models of care are described here in order of increasing complexity. Details on the advantages and challenges of the various models can be found in Table 1 [37].

**Table 1:** Possible models of care in geriatric oncology and their advantages and challenges

Model of care	Pathway	Advantages	Challenges
Consultative	Oncologist refers patient Reasons: GA and intervention recommendations, treatment recommendations GA performed by geriatrician and multidisciplinary team (consisting of allied health professionals and geriatric experts)	Geriatric/geriatric oncology expertise Recommendations from a multidisciplinary team	Physician buy-in need to refer One time visit No longitudinal follow-up Interventions often left to treating team Long visits: Limited no. of patients per clinic session Multiple visits and physicians for patients Need to maintain good communication in the team
Shared care	Oncologist refers patient Reasons: GA and intervention recommendations, treatment recommendations GA performed by geriatrician and/geriatric oncologist and multidisciplinary team Interdisciplinary meeting to review the results and care plan Geriatric oncology team collaborates with treating oncologist and provides concurrent care across the disease trajectory	Collaborative care through disease trajectory Geriatric/geriatric oncology expertise Interventions and multidisciplinary recommendations can be implemented over time	Physician buy-in needed to refer Visits may not be centralized Shortage of geriatricians Extra visits for the patient
Comprehensive	Geriatric oncologist is the treating oncologist throughout the patient’s disease trajectory No need for additional referrals GA performed Results and recommendations are reviewed with the patients Referrals to the multidisciplinary team	Geriatric oncology expertise throughout the treatment trajectory Convenience: One-stop shop (geriatrics and oncology)	Shortage of geriatric oncologists Complex patient population (limited no. of patients can be seen)
Resource limited alternatives	Option 1 Use a frailty screening tool to assess who needs a GA or who is vulnerable	Less time consuming For the use of geriatric screening tools no geriatric expertise necessary Less GAs need to be performed	Challenging to select the optimal screening tool and decide accurate cut-offs Screening tools are less accurate in identifying who may benefit from geriatric interventions, over- and under-treatment may be a consequence
	Option 2—Let other health care professionals without geriatric expertise perform a GA and use an existing intervention protocol to implement non-oncologic interventions	Can be used when there is a lack of geriatric expertise No additional time is required from the oncology team	Result of GA is not taken into account in oncologic treatment plan, so less effect on outcomes is expected

Table based on: Battisti and Efrat [37]

Within a consultative model, the oncology team refers patients to the geriatrics team for GA and recommendations on interventions and treatments. GA is performed by the geriatrician and the multidisciplinary team. In a shared-care model, the oncology teams refer patients to a geriatric medicine or a geriatric oncology team, but their care is shared by both teams. In a comprehensive model, the geriatric oncology team is the treating team. Therefore, this model does not involve any referrals to different teams as geriatric assessments and driven interventions are performed and implemented by the same team that is responsible for the oncologic treatment.

No evidence exists on superiority of any of the models or on who performs GA. However, it is important to have either specific geriatric expertise in the team, or to have a pre-defined intervention protocol on what to do if geriatric impairments are present [24]. Another key aspect of any model to achieve benefit is that the result of GA is taken into consideration when defining the oncologic treatment plan [24].

## Approach in resource-poor or time-limited settings

The gold standard to assess a patient’s fitness is a GA. However, if there is a lack of time or resources to perform a full assessment in all patients over a certain age, brief frailty screening instruments could be used to select those patients who may be most vulnerable and require a full GA [17]. Even in circumstances where a GA is not feasible, adding a screening tool to the routine assessment can predict which older adults are at risk of functional decline, toxicity or mortality [18, 38]. If resources are limited, but some is available, one of the less complex models described in the previous section can be chosen (Table 1).

Traditionally, GA is performed by a geriatrician, but as there are not enough geriatricians in most countries, other health professionals such as advanced practice nurses or physician associates could be trained to perform all or part of a GA. They could collaborate with geriatricians or use a pre-made intervention protocol. Examples of these protocols are available from GAP 70 + and GAIN studies [25, 26].

In even more resource-limited settings, self-administered GA tools may be considered, where patients or their caregivers help to provide information by answering questions as part of an assessment. These could be either on paper, or on a tablet or smartphone and will save time for the healthcare professionals [39, 40].

## Recommendations to policymakers to facilitate implementation of geriatric assessment

Clinical implementation in geriatric oncology is only feasible where there is support from both management at local hospital levels and policymakers on either national or international level. There are areas where further contribution from policymakers is necessary to enhance the implementation of geriatric oncology in clinical practice. Since policymakers are operating on various levels, depending on their position they may contribute differently.

For policymakers working on a local or regional level, a first step is to assess current care pathways to identify those aspects that don’t align with the most recent evidence on older adults with cancer. Advocating for better implementation in local, national and international guidelines may be useful, since a change in guidelines may be a first step towards changing care in clinical practice. Some international clinical practice guidelines have been written and may be used as a starting point [13, 41].

Policymakers working for global and national public funding organisations may aid in finding ways to fund research in wealthy and resource-limited settings. This is necessary if this fast growing population, typically underrepresented in traditional oncology research, is to be treated according to the best evidence-based approaches [7]. To aid researchers in geriatric oncology, the Cancer and Aging Research Group (CARG) was established. Its mission is to join researchers on this subject in a collaborative effort to design and implement clinical trials in this specific population [42].

Additionally, reimbursement strategies need to be developed for geriatric evaluation and geriatrician involvement, beyond the standard oncologic medicine reimbursement. This way proper personalized evaluation and right-sizing of therapy will be rewarded, which may promote workforce recruitment. Education and training will also aid cancer workforce upskilling around frailty and benefits of collaboration between oncologists and geriatric experts, as it is an important step to empower healthcare professionals to treat older adults with cancer more appropriately. Several educational and training opportunities are available both in high and low-middle income countries, such as the SIOG Advanced Course in Geriatric Oncology [43]. Furthermore, the American Society of Clinical Oncology (ASCO) and the European Society of Medical Oncology (ESMO) provided recommendations for core geriatric oncology training in the Global Curriculum in Medical Oncology [7, 44]. Policymakers involved in education or upskilling from medical professionals may take these options into consideration.

Since the availability of resources may vary substantially between and within countries, best practices and recommendations may be needed on what services could be implemented. The SIOG Public Policy committee was established in 2020 for advice on public policy.

## Conclusions

Older patients with cancer benefit from incorporating a multi-domain GA into decision making in their oncologic care trajectory. Clinical evidence exists on its benefits on outcomes such as toxicity rates, quality of life, mobility, patient centred-communication and healthcare utilisation. Furthermore, GA is cost-effective as it prevents inadequate treatment and therefore allows better access to and value of care, while reducing inappropriate use of expensive oncologic treatments reducing the costs of adverse events. A tipping point has been reached where the evidence base has been demonstrated, and cost-effectiveness is being realised. However, implementation in clinical practice is still limited. Now is the time for policymakers to embrace the gold standard of care and ensure that the older population has access to these vital services. Multiple options exist for both high and low resource settings and the geriatric oncology community is available to share best practices to enhance implementation against the background of an ageing population.

## Data Availability

Only publicly available data used.
